# HER2 alterations in non-small cell lung cancer (NSCLC): from biology and testing to advances in treatment modalities

**DOI:** 10.3389/fonc.2025.1624124

**Published:** 2025-06-20

**Authors:** Ahmed Ismail, Aakash Desai, Yanis Boumber

**Affiliations:** Section of Hematology/Oncology, Department of Medicine, The University of Alabama at Birmingham and O’Neal Comprehensive Cancer Center, Birmingham, AL, United States

**Keywords:** non-small cell lung cancer (NSCLC), ErbB2 (HER2), HER2 testing, targeted therapy, tyrosine kinase inhibitors (TKIs), antibody-drug conjugates (ADCs), immunotherapy

## Abstract

Lung cancer remains the leading cause of cancer-related deaths worldwide. Non-small cell lung cancer (NSCLC), which accounts for approximately 85% of all lung cancers, is a biologically diverse disease characterized by a wide range of molecular alterations. Among these, HER2 (human epidermal growth factor receptor 2, or Erb-B2 Receptor Tyrosine Kinase 2 (ERBB2)), a gene more commonly associated with breast cancer, has emerged as an important oncogenic driver in NSCLC, particularly within the adenocarcinoma subtype. HER2 alterations are notably more prevalent among non-smokers, with estimates suggesting that up to 50–80% of patients with HER2 mutations or amplifications have no smoking history. In our comprehensive review, we outline the molecular biology of HER2 in NSCLC, including distinctions between HER2 mutations, amplification, and overexpression, and we delve into the diverse diagnostic complexities. We also review NCCN guidelines and the performance of newer FDA-approved testing assays (such as Guardant360 and FoundationOne) in detecting HER2 alterations and circulating tumor DNA (ctDNA) as a tool for treatment response monitoring. Furthermore, we present updated clinical trial data for published HER2-targeted agents and explore ongoing clinical trials examining combinatorial therapies and next-generation HER2-targeted agents such as zongertinib, A166, ARX788, SHRA1811, and others. Given the rapid evolution in this field, our review offers a timely and comprehensive synthesis of the current state and future directions for HER2-altered NSCLC.

## Introduction

Lung cancer is the leading cause of cancer-related mortality worldwide. Non-small-cell lung cancer (NSCLC) is a heterogeneous disease driven by a broad spectrum of molecular alterations and represents 85% of all lung cancer (1, 2).

**Figure 1:**
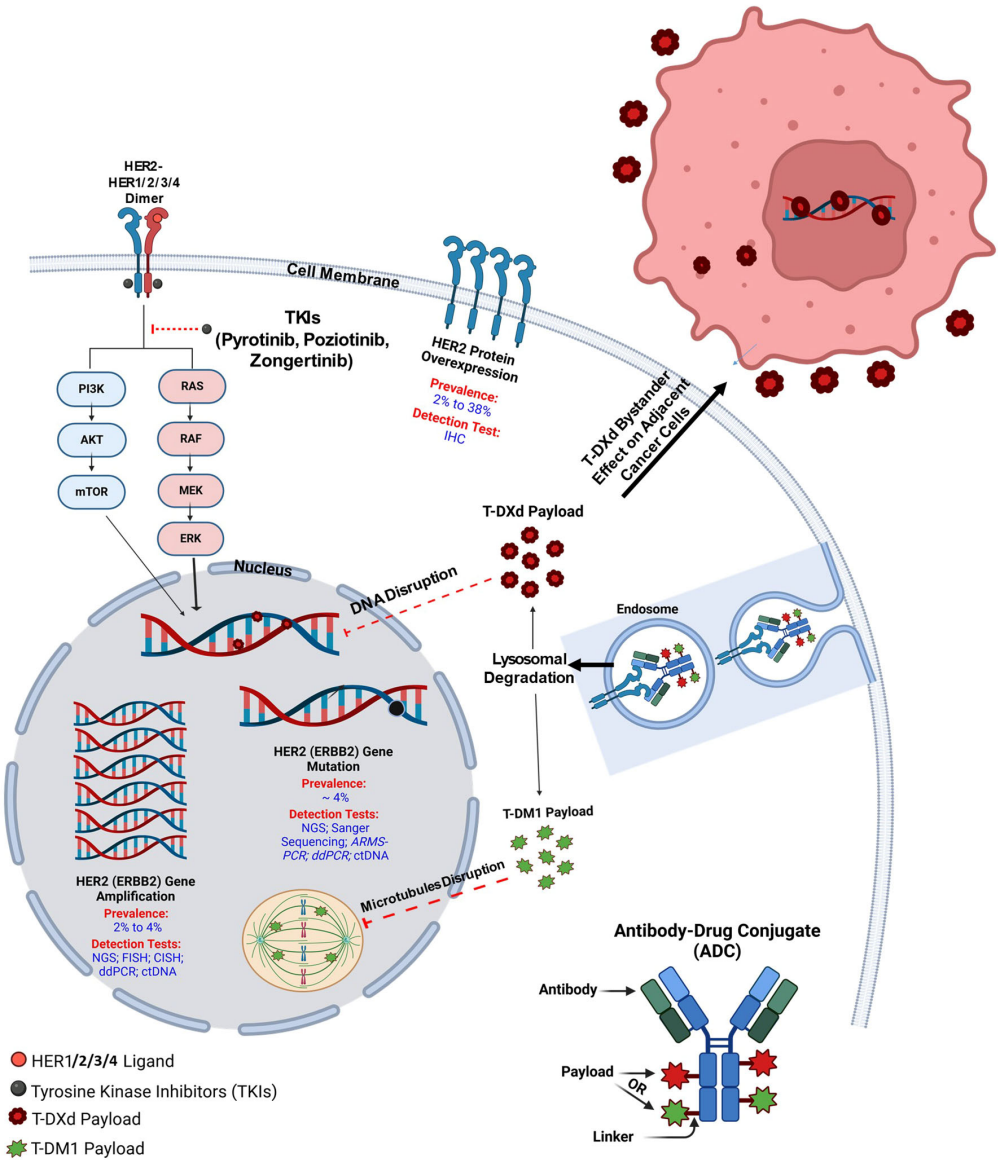
HER2 alterations and mechanism of action of ADCs and TKIs.

The human epidermal growth factor receptor 2 (HER2 or erbB-2/neu) is a well-known oncogene and an important biomarker in breast cancer, especially in HER2-positive subset, where several targeted agents have been approved in recent years (3). It is also a critical therapeutic target in gastroesophageal adenocarcinoma (GEA), where HER2 overexpression or amplification occurs in about 15–20% of cases (4). Trials have shown that adding trastuzumab to chemotherapy significantly improves survival in HER2-positive advanced GEA, establishing HER2 testing as standard care (5). Recently, HER2 has also been identified as an oncogene and therapeutic target in NSCLC, primarily in the lung adenocarcinoma subtype (6). HER2 is a transmembrane growth factor receptor with intrinsic tyrosine kinase activity, which can lead to unregulated cell growth, survival, and proliferation of cancer cells upon aberrant activation (7). HER2 alterations include protein overexpression, as well as gene amplification and mutations (**Figure 1**) (2). HER2 mutations in NSCLC were first detected in 2004 (8) and occur in about 4% of NSCLC patients, with most mutations commonly found within exon 20 of the HER2 gene (9). Although the evidence to support routine HER2 testing in NSCLC is limited, the United States National Comprehensive Cancer Network (NCCN) 2025 guidelines recommend testing for rare oncogenic driver alterations (including HER2 mutations) for better therapeutic decision-making.

For two decades, platinum-based chemotherapy, and since 2018, with the addition of immunotherapy, chemoimmunotherapy has been the standard treatment for stage IV NSCLC patients (10). Initial development of HER2-targeted monoclonal antibodies (trastuzumab and pertuzumab) and older pan-HER tyrosine kinase inhibitors (TKIs), such as afatinib, did not yield promising antitumor activity in NSCLC patients with HER2 alterations. However, recently, novel selective HER2 TKIs, such as poziotinib, have shown more encouraging antitumor effects than previous anti-HER2 agents have (11, 12). Additionally, in August 2022, the FDA granted accelerated approval to trastuzumab-deruxtecan (T-DXd) antibody-drug conjugate (ADC) based on impressive DESTINY-Lung02 trial results, making it the first approved targeted therapy for HER2-mutant NSCLC (13).

## HER2 biology

HER2 (ERBB2) is a member of the ERBB-receptor family, which also includes ERBB1 (EGFR), ERBB3, and ERBB4. It is a transmembrane growth factor receptor with intrinsic tyrosine kinase activity, encoded by the ERBB2 gene on chromosome 17q12, and is critical for cancer cell growth and survival (14). Structurally, HER2 consists of an extracellular ligand-binding domain, a transmembrane region, and an intracellular tyrosine kinase domain (15). While HER2 has no identified ligand, unlike other ERBB family members, it is activated through dimerization with other ERBB receptors, serving as a preferred partner for other ERBB family members, such as HER1 (EGFR) or HER3, enhancing their signaling efficiency. Such activation initiates downstream signaling through the PI3K/AKT/mTOR and RAS/RAF/MEK/ERK pathways (**Figure 1**), promoting cell proliferation and inhibiting apoptosis (16). HER2 alterations in NSCLC include gene mutations (most commonly small insertions or duplications in exon 20) (17), gene amplification (an increase in HER2 copy number) (18), and protein overexpression. These alterations can occur independently and have different biological consequences. Mutations often lead to constitutive activation of the HER2 tyrosine kinase, while amplification results in increased downstream signaling due to higher receptor density. Overexpression reflects elevated HER2 protein levels, typically measured by immunohistochemistry (IHC), and is not always driven by gene amplification in NSCLC. Based on that, overexpression or amplification of HER2, frequently observed in breast and gastric cancers, correlates with aggressive tumor behavior and poor prognosis (19). In addition, in various cancer types like NSCLC, breast, colorectal, bladder, gastric, and esophageal cancers, HER2 mutations, particularly exon 20 insertions, lead to constitutive tyrosine kinase activation, driving oncogenesis without ligand stimulation (3, 5, 20–23).

### HER2 mutations in NSCLC

HER2 gene mutations in NSCLC represent a distinct molecular subset, occurring in approximately 4% of cases, with a higher prevalence in adenocarcinoma histology, non-smokers, and females (24). Notably, HER2-altered NSCLC is more commonly found in never-smokers compared to the general NSCLC population. Studies indicate that 60-80% of patients with HER2 mutations or amplifications in NSCLC have no history of smoking (25–27). The prevalence of HER2 mutations in non-smokers is notably higher compared to some other oncogenic driver mutations, such as KRAS, which are more frequently associated with smoking. HER2 mutations play a critical role in tumorigenesis by driving a constitutive activation of downstream signaling pathways like PI3K/AKT/mTOR and RAS/RAF/MEK/ERK, which regulate cell proliferation, survival, and growth (15). HER2 mutations in NSCLC are predominantly somatic and located within the tyrosine kinase domain, with exon 20 insertions being the most common type. The most frequent HER2 mutation is A775_G776 insYVMA. It is an insertion of four amino acids Tyrosine (Y), Valine (V), Methionine (M), and Alanine (A) between Alanine at position 775 (A775) and Glycine at position 776 (G776) on exon 20. A775_G776insYVMA, accounts for approximately 34% of all HER2 mutations and results in constitutive receptor activation independent of ligand binding (28). Other mutations like G776delinsVC and G778_P780insGSP also occur in the tyrosine kinase domain, also driving oncogenic signaling (24).

HER2 mutations are usually mutually exclusive with other driver mutations like EGFR, ALK, or ROS1, suggesting a specific HER2-driven biology HER2-mutant NSCLC (17). This exclusivity highlights the necessity of targeted molecular testing for HER2 mutations, especially in patients with no other driver mutations. In addition to exon 20 insertions, other rarer HER2 mutations, including those affecting the extracellular and transmembrane domains, have been identified, such as I655V, P122L, and S310F. Although rare, these mutations contribute to tumor heterogeneity and may influence clinical behavior and therapeutic response (24).

Of note, HER2 mutations in NSCLC are associated with an aggressive disease course, including a higher likelihood of brain metastases, particularly in tumors harboring YVMA mutations (28). This predisposition underscores the need for comprehensive CNS imaging in this patient population. Furthermore, HER2 mutations pose unique challenges in treatment due to their heterogeneity and varying responses to targeted therapies. Traditional HER2-directed therapies, such as trastuzumab and pertuzumab, which are highly effective in HER2-amplified breast cancer, have shown limited efficacy in HER2-mutant NSCLC (29, 30). Tyrosine kinase inhibitors (TKIs) such as neratinib, afatinib, and poziotinib have demonstrated promise, with poziotinib showing activity against exon 20 insertion mutations (12). However, the clinical response remains inconsistent, reflecting the complexity of HER2 biology in NSCLC.

HER2 mutations define a unique subset of NSCLC with distinct molecular characteristics and clinical implications. Comprehensive molecular profiling is essential to identify these mutations and tailor treatment strategies. With the advent of new-targeted therapies explicitly targeting HER2 mutations, the prognosis for patients with HER2-mutant NSCLC is improving. However, challenges such as resistance mechanisms and disease heterogeneity remain active research areas. These efforts aim to optimize patient outcomes and advance the understanding of HER2-driven lung cancer biology.

### HER2 amplification in NSCLC

HER2 amplification is less prevalent in NSCLC compared to other malignancies, such as breast cancer. In NSCLC, HER2 amplification represents a distinct molecular subset, accounting for only 2% to 4% of cases, primarily adenocarcinoma. It is notably more prevalent in males and smokers (31). HER2 amplification leads to increased HER2 gene copies, resulting in overexpression of the HER2 protein on the cell surface, which promotes tumorigenesis by activating downstream signaling pathways (31).

HER2 amplification has been typically detected using fluorescence *in situ* hybridization (FISH), which identifies the increased copy number of the HER2 gene. Generally, it is defined as HER2/CEP17 ≥ 2.0 using FISH. Next-generation sequencing (NGS) is a new and now more commonly used alternative to FISH to detect HER2 amplification, providing additional comprehensive genomic profiling. However, routine testing for HER2 amplification in all NSCLC patients is currently not recommended due to limited evidence supporting its role as a predictive biomarker for targeted therapies. Testing is more commonly considered in clinical trials or when exploring treatment resistance mechanisms, such as the EGFR TKIs (18). However, the rapid expansion of molecular tests with many platforms now allows routine detection of HER2-amplified NSCLC subsets in clinical practice.

The clinical significance of HER2 amplification in NSCLC is an area of ongoing research. Some studies suggest that HER2 amplification may contribute to resistance against EGFR TKIs, highlighting the need for alternative therapeutic strategies in this subset of patients (32). It has been reported that HER2 amplification in NSCLC is associated with aggressive tumor characteristics, including larger tumor size, an increased likelihood of pleural metastases, and a higher rate of lympho-vascular invasion. However, its prognostic and predictive significance in NSCLC remains uncertain and requires further investigation (6). Additionally, despite the availability of HER2-targeted therapies in other cancers, such as trastuzumab in breast cancer, their efficacy in NSCLC patients with HER2 amplification has been limited. This underscores the necessity for continued research to develop effective treatments for this specific molecular HER2 alteration.

### HER2 overexpression in NSCLC

HER2 overexpression in NSCLC is a common molecular alteration, with reported prevalence rates varying widely from 2% to 38% (24). This variability is attributed mainly to differences in detection methodologies and scoring criteria. IHC is the primary technique to assess HER2 protein levels in tumor tissues. However, the lack of standardized scoring systems for HER2 overexpression in NSCLC has led to inconsistencies in reporting and interpretation (31). Like HER2 amplification, overexpression is more prevalent in males and in smokers (31). In NSCLC, HER2 overexpression is often linked to polysomy rather than HER2 amplification, unlike in breast cancer, where HER2 amplification is strongly associated with HER2 expression. Polysomy is a HER2 gene copy number exceeding 5 or 6 with a HER2/CEP17 ratio of less than 2 (33).

The most commonly used scoring system depends on the tumor type, whether breast or non-breast according to the American Society of Clinical Oncology/College of American Pathologists (ASCO/CAP).

For Breast Cancer, ASCO/CAP HER2 IHC Scoring (34): 0: No staining or incomplete membrane staining in <10% of tumor cells,

1+: Faint/barely perceptible incomplete membrane staining in >10% of tumor cells,
2+: Weak to moderate complete membrane staining in >10% of tumor cells, and 3+: Uniform, intense circumferential membrane staining in >10% of tumor cells.

For non-breast cancer tumors, ASCO/CAP Gastric Cancer HER2 IHC Scoring is usually applied:

0 or 1+: Negative, regardless of the number of cells staining,
2+: Equivocal if ≥5 tumor cells exhibit staining; otherwise, negative,
3+: Positive if ≥5 tumor cells exhibit staining; otherwise, negative.

HER2 overexpression is more frequently observed in adenocarcinoma subtypes of NSCLC than squamous cell carcinomas. Within adenocarcinomas, overexpression has been notably associated with papillary-predominant histology, suggesting a potential link between HER2 status and specific morphological features (18). A meta-analysis of 6,135 lung cancer patients indicated that HER2 overexpression determined by IHC is a marker of poor prognosis in NSCLC (HR 1.48; 95% CI: 1.22–1.80), especially in small cell lung cancer, lung adenocarcinoma, and early-stage NSCLC patients (35). Notably, the types of antibodies used for IHC to detect HER2 varied across these studies. The commonly utilized antibodies included CB11 and TAB250 (monoclonal antibodies), and other polyclonal antibodies targeting HER2 were used in some studies (35).

## HER2 molecular profiling methods in NSCLC

Detecting HER2 (ERBB2) alterations in NSCLC is crucial for guiding targeted therapies and improving patient outcomes. Various methodologies are employed to identify HER2 mutations, amplifications, and protein overexpression, each with distinct advantages and limitations.

### HER2 mutations detection

*Next-generation sequencing (NGS)* (36) represents a commonly used high-throughput sequencing approach, enabling the detection of a wide range of genetic alterations with high sensitivity and specificity. NGS is considered the most comprehensive approach for identifying HER2 mutations in NSCLC and is the preferred method for detecting HER2 mutations when available.

*Sanger sequencing* (37) is a traditional method that sequences DNA to identify specific mutations. While accurate, it is less sensitive, may not detect low-frequency mutations, and is rarely used now.

*Amplification Refractory Mutation System-PCR (ARMS-PCR)* (38) is a sensitive method to detect known mutations, including those in HER2. It is cost-effective but limited to predefined mutations.

*Droplet Digital PCR (ddPCR)* (39) precisely quantifies DNA mutations, offering high sensitivity for detecting low-abundance mutations. It is beneficial for monitoring known mutations.

### HER2 amplification detection

*Next-generation sequencing (NGS)* (36), as previously mentioned, represents a high-throughput sequencing approach, enabling the detection of a wide range of genetic alterations, including HER2 amplification, with high sensitivity and specificity. It is the most common test used nowadays and the preferred method for detecting HER2 amplifications (18).

*Fluorescence In Situ Hybridization (FISH)* (40) is considered the gold standard for detecting HER2 amplification. FISH uses fluorescent probes to visualize HER2 gene copies within the tumor cells. It provides high sensitivity and specificity.

*Chromogenic In Situ Hybridization (CISH)* (41) is similar to FISH but utilizes chromogenic substrates, allowing evaluation under a standard bright-field microscope. CISH offers comparable sensitivity and specificity to FISH and has the advantage of permanent staining.

*Multiplex Droplet Digital PCR (ddPCR)* (42) enables simultaneous detection of multiple gene amplifications, including HER2, with high precision. This method is beneficial for analyzing limited tissue samples.

### HER2 protein overexpression detection

*Immunohistochemistry (IHC)* (43) utilizes antibodies to detect HER2 protein expression in tumor tissues. IHC is widely used due to its accessibility and ability to provide information on protein localization within the tissue context. However, standardization of scoring criteria in NSCLC is necessary to ensure consistent results. Commonly used anti-HER2 IHC antibodies can be either monoclonal or polyclonal. Monoclonal antibodies are preferred for their specificity and better reproducibility. However, polyclonal antibodies have the advantage of higher sensitivity (43). Commonly used monoclonal anti-HER2 IHC antibodies include CB11 and TAB250. Additionally, SP3 is a rabbit monoclonal antibody commonly used in research and clinical diagnostics. Notably, 4B5 (Ventana PATHWAY anti-HER2/neu) is a widely used rabbit monoclonal antibody approved for HER2 testing in breast and gastric cancers, also applied in NSCLC (44).

### Newer tests for HER2 detection

*Circulating Tumor DNA (ctDNA) Analysis* (45) detects HER2 mutations and amplifications in blood samples using techniques like NGS or ddPCR. This novel approach allows for real-time monitoring of tumor genetics and treatment response. A liquid biopsy (46) (ctDNA testing) can be obtained in cases where tumor tissue is unavailable or insufficient. This minimally invasive test offers an alternative to the standard tests. The most common clinically used liquid biopsy assays include Guardant, Tempus, Caris, and FoundationOne. Although ctDNA analysis is an emerging minimally invasive option, its availability may vary by institution and may sometimes require confirmation through tissue-based testing, using NGS or another method (like IHC/FISH).

*Circulating Tumor Cells (CTCs) Detection* (47) isolates and analyzes tumor cells circulating in the bloodstream. Methods include immune-magnetic separation using antibodies against tumor-specific HER2 markers. CTC analysis can provide insights into tumor biology and metastatic potential. However, it is mainly considered an investigational test that requires more confirmation using other tests and is used for research purposes only.

### Considerations and challenges

Selecting the appropriate detection method depends on various factors, including sample availability, the specific type of HER2 alteration, and the clinical context. NGS offers comprehensive profiling but may be limited by cost and accessibility. IHC and FISH are more widely available but require standardized protocols to ensure accuracy. Liquid biopsies present a promising non-invasive option but may have limitations in sensitivity compared to tissue-based methods. These factors should be considered to detect HER2 alterations in NSCLC accurately, which can be vital for personalized treatment strategies and improved patient care.

### Clinical guidelines for HER2 testing

In early and locally advanced NSCLCs, NCCN (Version 3, 2025) (48) states that testing for certain biomarkers is recommended for eligible patients with resectable early-stage and locally advanced NSCLCs, such as larger tumors. Biomarker tests typically include the most common ones, including PD-L1 level, EGFR (ERBB1) mutations, and ALK gene rearrangement.

In advanced or metastatic NSCLCs, complete genotyping for EGFR, KRAS, ALK, ROS1, BRAF, NTRK1/2/3, MET, RET, ERBB2 (HER2), and NRG1 via biopsy and/or plasma testing should be carried out. Combinations of tissue and plasma testing, either concurrently or in sequence, are acceptable. Concurrent testing can improve the time to test results and should be preferred. Negative results, meaning an absence of definitive driver mutation by one method, suggest using a complementary technique. Testing should include broad molecular profiling, which is a key component of improving the care of patients with NSCLC. The NCCN NSCLC Guidelines NCCN panel strongly advises broader molecular profiling to identify rare driver mutations for which effective drugs may already be available or to counsel patients regarding the availability of clinical trials appropriately. Broad molecular profiling is defined as molecular testing that identifies all aberrations in EGFR, KRAS, ALK, ROS1, BRAF, NTRK1/2/3, MET, RET, ERBB2 (HER2), NRG1, and PD-L1 in either a single assay or a combination of a limited number of assays. NCCN also recommends that testing for HER2 IHC be done at some point during progression, with a balance between timing and tissue conservation. In adenocarcinoma, large cell carcinoma, and NSCLC not otherwise specified (NOS), HER2 testing, along with the other drivers as part of broad molecular profiling, should be carried out routinely on all patients. In squamous cell carcinoma, such tests should also be considered on a case-by-case basis.

## HER2-targeted treatment strategies

Within the past few years, several studies on HER2 Tyrosine Kinase Inhibitors (TKIs), Immune Checkpoint Inhibitors (ICIs), Monoclonal Antibodies, and Antibody-Drug Conjugates (ADCs) have emerged for patients with HER2-altered NSCLC.

### Pan-HER TKIs

Early attempts to target HER2 mutations were made with pan-HER TKIs. This family included Dual EGFR/HER2 TKIs such as afatinib and other irreversible pan-HER TKIs like dacomitinib, neratinib, or pyrotinib (**Table 1**). However, these drugs have shown little activity against HER2-mutant refractory NSCLC in small phase II studies.

**Table 1:** Clinical outcomes of Pan-HER and selective HER2 TKIs in HER2-mutant NSCLC.

Agent	Study/Population	ORR %	DCR %	mPFS (months)	mOS (months)	Notable AEs / Grade ≥3 (%)
Afatinib	Phase II (n=13, pretreated)	–	53.8% at 12 weeks	3.7	12.9	Diarrhea, rash, mucositis
	NPU (n=28, pretreated)	19	69	–	–	Diarrhea (42.9%), rash (35.7%)
	Phase II (n=32, pretreated)	0	–	2.76	10.02	Not specified
Dacomitinib	Phase II (HER2-mutant or amplified, n=30)	12 vs 0	–	–	9 in HER2 mutant	Diarrhea, fatigue, mucositis
Neratinib	Basket trial (Subgroup with HER2-mutant Lung Cancer, n=26)	3.8	–	5.5	–	Diarrhea (54%), nausea
Poziotinib	Phase II (n=90, pretreated)	27.8	70	5.5 (mDoR of 5.1 months)	–	Grade ≥3 TRAEs: 78.9%, rash, diarrhea, stomatitis
	Phase II (n=80, treatment-naive)	39	73	5.6 (mDoR of 5.7 months)	–	Grade ≥3 TRAEs: 79%, less dose reductions vs. QD
Pyrotinib	Phase II (n=60, pretreated)	30	–	6.9	14.4	Grade ≥3 TRAEs: 40% (diarrhea most common)
	Phase II (n=78, pretreated and naive)	19.2	–	5.6 (8.9 in naive) (mDoR of 9.9 months)	10.5	Similar toxicity
	Phase II Pyrotinib + apatinib (pretreated, n=33)	51.5	93.9	6.9 (mDoR of 6.0 months)	14.8	Grade ≥3: 12.1%
	Phase 1b Pyrotinib 240 mg or 320 mg + Inetetamab (n=48)	0 vs 36.8	66.7 vs 85.4	5.5 in the 320 mg group	–	Mostly diarrhea, rash
Tarloxotinib	Phase II (cohort B, n=11)	22	67	–	–	QTc prolongation (34.8%), rash, diarrhea
Mobocertinib	Phase Ia/Ib Mobocertinib + T-DM1 (n=13)	53.8	84.6	6.1	–	Diarrhea, nausea, fatigue
	EXCLAIM-2 Trial	–	–	–	–	Withdrawn (failed to beat chemotherapy)
Zongertinib	Beamion LUNG-1 Phase I (cohort 1, n=75)	71	93	6-mo PFS: 69% (6-mo DOR of 73%)	–	Mostly Grade 1–2 AEs; no ILD; diarrhea (51%), rash (27%)
	Beamion LUNG-1 Phase Ib	72	95.5	–	–	–

[References: 11, 50–67].

#### Afatinib

A prospective, open-label phase II trial explored the potential of afatinib to control disease in pretreated patients with advanced NSCLC harboring HER2 exon 20 mutations. Thirteen patients entered the trial and were treated with afatinib 40 mg/day. Seven patients (53.8%) achieved disease control at 12 weeks. However, progression was documented for all patients, with a median progression-free survival (mPFS) of 3.7 months and a median overall survival (mOS) of 12.9 months (49). Another global named patient use (NPU) program assessed the activity of afatinib in 28 heavily pretreated patients with HER2-mutant advanced NSCLC. The results of this NPU showed an ORR of 19% (3 of 16 patients with response data achieved a partial response) and a disease control rate (DCR) of 69%. Notably, in a subgroup analysis of patients with p.A775_G776insYVMA insertion mutation, ORR was 33% (2 PR), and the DCR was 100% (4 SD and 2 PR). This analysis suggested that identifying specific subgroups with certain mutations, such as p.A775_G776insYVMA in exon 20, could help optimize outcomes with HER2-targeted treatment (50). Another phase II trial assessed afatinib in patients previously treated with chemotherapy with advanced EGFR TKI-naïve HER2-mutant NSCLC. The results showed no patients achieving an objective response with an mPFS of 2.76 months and mOS of 10.02 months. Consequently, this study found no clinical benefit of afatinib for EGFR TKI-naïve patients with HER2-mutant NSCLC (51).

#### Dacomitinib

A phase II trial assessed dacomitinib in 30 patients with HER2-mutant (ex20ins) or amplified stage IIIB/IV lung cancers. In the HER2-mutant cohort (26 patients), ORR was 12%, and mOS was 9 months. Notably, in the HER2-amplified cohort (4 patients), ORR was 0% (52).

#### Neratinib

A global, multicenter, multi-histology basket trial enrolled 141 pretreated patients with HER2 and HER3-mutant cancers to assess Neratinib treatment. In a subgroup analysis of 26 HER2-mutant lung cancer patients (predominantly ex20ins), ORR was 3.8%, and mPFS was 5.5 months (53).

#### Pyrotinib

Pyrotinib is a small, irreversible inhibitor of the EGFR, HER2, and HER4 receptors, approved as a second-line treatment for advanced HER2-positive breast cancer.

A prospective, multicenter, single-arm phase II trial of 60 platinum-pretreated advanced NSCLC patients with HER2 mutations receiving pyrotinib demonstrated favorable outcomes. An ORR of 30%, mPFS of 6.9 months, and mOS of 14.4 months were reported. The subgroup analysis of this trial indicated that all patients with various HER2 mutation subtypes, regardless of whether they had brain metastases, could benefit from pyrotinib. However, 98.3% of patients experienced TRAEs, including 28.3% in grade 3 or 4, commonly presenting as diarrhea (20%). Dose interruptions of 21.7% and treatment discontinuations in 1.7% of patients were reported (54). Another prospective Phase II clinical trial involving 78 patients with HER2-mutant advanced NSCLC utilized pyrotinib as first-line or later-line treatment. The ORR was 19.2%, with an mPFS of 5.6 months, mDoR of 9.9 months, and mOS of 10.5 months. Notably, treatment-naive patients (n = 23) exhibited a superior mPFS of 8.9 months (55). Furthermore, a phase III, randomized, open-label, multi-center study is currently underway to evaluate the efficacy and safety of pyrotinib compared to Docetaxel in patients with advanced non-squamous NSCLC who have a HER2 ex20 mutation and have progressed on or after platinum-based chemotherapy. This phase III study aims to recruit 150 eligible subjects to be randomized in a 2:1 ratio (study treatment arm: Control Arm = 100: 50 subjects) to receive either pyrotinib or docetaxel monotherapy (NCT04447118).

In recent years, multiple studies have been conducted to evaluate using pyrotinib in combination with other agents rather than as monotherapy. For example, a prospective phase II study (PATHER2) enrolled 33 advanced NSCLC patients with HER2 alterations (31 with HER2 mutations) who had failed one or more lines of therapy and examined the combination of pyrotinib with apatinib (an anti-angiogenic TKI). The ORR was 51.5%, DCR was 93.9%, mDoR was 6.0 months, mPFS was 6.9 months, and mOS was 14.8 months. All patients experienced at least one TRAE, regardless of the grade. However, only 12.1% of patients had TRAEs of grade ≥ 3 (56). These results suggest that this combination regimen is likely superior to pyrotinib monotherapy in terms of ORR and safety.

Another study assessed the combination of pyrotinib with the HER2-targeted monoclonal antibody inetetamab (NCT05016544). This Phase 1b dose-escalation, dose-expansion trial evaluated the combination’s safety and efficacy in patients with advanced HER2-mutant NSCLC, enrolling 48 patients. Among patients receiving the inetetamab with pyrotinib 240mg, the confirmed ORR was 0%, and the DCR was 66.7%. However, in patients receiving inetetamab with pyrotinib 320mg, the study reported a confirmed ORR of 36.8%, a DCR of 85.4%, and an mPFS of 5.5 months (95% CI: 4.4–8.6 months). Subgroup analyses were conducted in the group of patients receiving inetetamab with pyrotinib 320mg. Notably, patients receiving inetetamab with pyrotinib as first‐line treatment showed better ORR than patients with prior therapies (40.9% [9/22] vs. 10.5% [2/19], p=0.038). However, both mPFS and DCR were similar in both groups. The mPFS was 5.5 vs. 5.5 months, p=0.81, respectively (first-line and prior therapies), and DCR was 86.4% [19/22] vs. 84.2% [16/19], p=1. The combination therapy demonstrated acceptable safety and antitumor activity in this patient population.

Other ongoing studies include a combination of pyrotinib with pemetrexed and carboplatin (NCT04706949), or PD-1 inhibitors (NCT04144569).

### Selective HER2 TKIs

More selective and novel pan-HER2 TKIs have recently emerged and have promise to improve outcomes in HER2-mutant NSCLC. Such drugs include poziotinib, tarloxotinib, mobocertinib, zongertinib, and others (**Table 1**) (11, 50–67). Additionally, multiple ongoing clinical trials have been conducted to check various selective TKIs (**Table 2**) (64, 65, 68–71). Here, we discuss some of these drugs in further detail.

**Table 2:** Ongoing clinical trials of selective HER2 TKIs in HER2-Altered NSCLC.

Drug name	NCT number	Phase	Study type	NSCLC population	Main HER2 alteration studied	Dosing regimen	Primary endpoint(s)	CNS/BM activity studied	Prior lines of therapy	Key outcomes (if available)
Zongertinib (BI 1810631)	NCT04886804	1a/1b	Open-label, dose-escalation, and expansion study	Advanced or metastatic NSCLC	HER2 mutations and amplifications	Oral, 120 mg once daily	Safety, ORR	Yes	Pre-treated	ORR 71%; DCR 93%; mDoR 8.5 months
BAY 2927088	NCT05099172	1/2	Open-label, first-in-human, dose-escalation, and expansion study	Advanced NSCLC	HER2 mutations	Oral, 20 mg twice daily	Safety, ORR	Not specified	Pre-treated	ORR 72.1%; mDoR 8.7 months; mPFS 7.5 months
NVL-330	NCT06151574	1a/1b	Open-label, dose-escalation, and expansion study	Advanced or metastatic NSCLC	HER2 alterations	Not specified	Safety, RP2D determination, preliminary anti-tumor activity	Yes	Pre-treated	No results reported yet
Pyrotinib	NCT04447118	3	Randomized, open-label, multicenter study	Advanced non-squamous NSCLC	HER2 exon 20 mutations	400 mg once daily	PFS, OS	Not specified	Post-platinum-based chemotherapy	No results reported yet
BAY 2927088	NCT05650879	3	Open-label, randomized study	Previously untreated advanced NSCLC	HER2-activating mutations	Oral, twice daily	PFS, OS	Not specified	Treatment-naïve	No results reported yet

[References: 64, 65, 68–71].

#### Poziotinib

Poziotinib is a novel small-sized, covalent, and irreversible dual EGFR/HER2 receptor inhibitor that helps to overcome the steric hindrance effect caused by HER2 exon 20 insertions (ex20ins). *In vitro* and patient-derived xenograft (PDX) preclinical models showed favorable preliminary data. In models with HER2 exon 20 mutant NSCLC, poziotinib appeared to be more effective than other pan-HER TKIs (72, 73), demonstrating an average half-maximal inhibitory concentration (IC50) value of 1.9 nM in Ba/F3 cell lines, making it more potent than osimertinib and afatinib by 200 times and 6 times, respectively.

In a multi-center and multi-cohort phase II study (ZENITH20) designed to assess poziotinib monotherapy in NSCLC, 90 pretreated patients with HER2 ex20 insertions received a daily (QD) dose of 16 mg of poziotinib. The ORR and DCR were 27.8% and 70.0%, respectively, with an mPFS of 5.5 months and a Median Duration of Response (mDoR) of 5.1 months. In this study (ZENITH20), Treatment-Related Adverse Effects (TRAEs), regardless of severity, were noted in 97.8% of patients. Grade 3 TRAEs were reported in 71 patients (78.9%), while grade 4 TRAEs and grade 5 TRAEs were documented in 4 and 1 patients (4.4% and 1.1%), respectively. Dose reduction occurred in 76.7% of patients, while discontinuation happened in 13.3% (58). A similar Phase II trial involving a smaller patient group (n = 30) investigated poziotinib (16 mg QD) in pretreated NSCLC with HER2 ex20 mutations. It yielded consistent results regarding the efficacy and safety of poziotinib (11).

Another multi-center, multi-cohort, open-label phase II trial (ZENITH20-C4) involved 80 treatment-naive patients with HER2-mutant NSCLC, assessing poziotinib at 16 mg QD (n = 47) and 8 mg BID (n = 33). The combined ORR was 39%, DCR was 73%, tumor reduction was 80%, mPFS was 5.6 months, and mDoR was 5.7 months. Moreover, the twice-daily regimen demonstrated a slightly lower incidence of dose reductions (70% vs. 79%), dose interruptions (85% vs. 89%), and overall TRAEs (97% vs. 100%) when compared to the once-daily regimen. However, grade ≥ 3 TRAEs were somewhat higher in the BID group (79% vs. 72%) (59).

Although poziotinib has demonstrated moderate efficacy in HER2-mutant NSCLC, the high risk of TRAEs, primarily those graded ≥ 3, raises significant concerns and limits further clinical development.

#### Tarloxotinib

Tarloxotinib is a small molecule hypoxia-activated prodrug that acts as a HER kinase inhibitor, mainly EGFR and HER2, and an NRG1 fusion inhibitor, releasing a potent irreversible active metabolite (Tarloxotinib-E) in hypoxic conditions.

RAIN-701 is a phase II trial that is currently recruiting chemotherapy-pretreated NSCLC patients harboring EGFR exon 20 insertions or HER2 mutations, as well as patients with any solid tumor and NRG1, EGFR, HER2, or HER4 fusions. Tarloxotinib is administered at an IV dose of 150mg/m^2^ weekly. In a small HER2-mutant NSCLC cohort B (n = 11), 44% of patients showed tumor reduction, 22% partial response (PR), and 44% stable disease (SD), resulting in a DCR of 67% in nine assessable patients. Most treatment-emergent adverse events (TEAEs) were grade 1 or 2. However, the reported grade 3 TEAEs included prolonged QTc (34.8%), rash (4.3%), diarrhea (4.3%), and increased ALT (4.3%). Additionally, 21.7% of patients required dose reductions, and 4.3% discontinued Tarloxotinib (60).

#### Mobocertinib

Mobocertinib is an oral TKI with potent, selective preclinical activity against activating EGFR and HER2 mutations, including exon 20 insertions. Mobocertinib has demonstrated antitumor activity in a phase I/II trial involving advanced NSCLC patients with EGFR exon 20 insertion (74, 75). In May 2024, a phase Ia/Ib trial on the safety and efficacy of Mobocertinib in combination with T-DM1 for patients with HER2-mutant solid tumors. In a subgroup analysis of 13 pts with HER2-mutant NSCLC, including those who had developed resistance to trastuzumab-deruxtecan, the ORR was 53.8%, the DCR was 84.6%, and mPFS was 6.1 months (95% CI 2.9, 6.3) (61).

Despite these results, the EXCLAIM-2 trial (NCT04129502) showed that mobocertinib was not superior to platinum-based chemotherapy for EGFR ex20ins NSCLC, leading to its voluntary withdrawal in October 2023 due to comparable efficacy (62, 63).

#### Zongertinib

Zongertinib is an investigational, irreversible TKI that selectively inhibits HER2 and spares EGFR to limit toxicities associated with EGFR inhibition. The phase I, Beamion LUNG-1 trial (NCT04886804), was an open-label, dose-escalation, and expansion study evaluating zongertinib monotherapy in patients with advanced or metastatic solid tumors harboring HER2 alterations that are refractory to or unsuitable for standard treatments. Findings from cohort 1 (n = 75) demonstrated an ORR of 71%, DCR of 93% with six-month PFS, and DOR rates of 69% and 73%, respectively. Safety data from the trial showed that adverse effects (AEs) led to low rates of dose reductions (5%) and treatment discontinuations (3%). Most TRAEs were mild; the most common any-grade TRAEs were diarrhea (51%) and rash (27%). Only one patient experienced grade 3 or higher TRAEs. Notably, no treatment-related interstitial lung disease was reported (64, 65).

In phase 1b of the study, patients with HER2-mutant advanced/metastatic NSCLC who are refractory to or unsuitable for standard treatments across five global cohorts are recruited. As of May 2024, efficacy analysis showed a confirmed ORR of 72.0% and a DCR of 95.5% in the entire patient population, with 2.3% achieving a CR and 69.7% achieving a PR. The duration of response and progression-free survival rate data remains immature, with 63.2% of responding patients still on treatment (66, 67).

Notably, in February 2025, the FDA granted priority review to its new drug application for Zongertinib (BI 1810631) for treating adult patients with unresectable or metastatic NSCLC whose tumors have HER2 mutations and who have received prior systemic therapy. Zongertinib is also under evaluation in the phase 3 Beamion LUNG-2 trial (NCT06151574), a randomized, active-controlled, first-line study comparing the agent with the standard of care in patients with unresectable or metastatic non-squamous NSCLC harboring HER2 mutations.

### Immune checkpoint inhibitors

Immune checkpoint inhibitors (ICIs) are a standard treatment for advanced NSCLC without oncogenic drivers. However, their effectiveness in patients with oncogene-driven NSCLC remains limited, mainly due to lower PD-L1 expression and low tumor mutation burden (TMB) (76).

In 2018, Lai et al. evaluated the efficacy of ICI as a monotherapy in a retrospective study in patients with advanced HER2-mutant NSCLC who received nivolumab, pembrolizumab, or other ICIs. The study showed an ORR of 12%, mPFS of 1.9 months, and OS of 10.4 months (77). Such results were comparable to other studies. In a study in 2019, smokers with HER2-mutant NSCLC (n=13) had a higher mPFS vs. nonsmokers (n=14) (3.4 months vs. 2.0 months) (78). Another study in 2020 looked at patients who had received one previous treatment, and the mPFS of 23 patients with HER2-altered NSCLC was 2.2 months (79).

In contrast, ICIs in combination with chemotherapy, Saalfeld et al. assessed the pembrolizumab-based immunochemotherapy first-line combination in HER2-mutated NSCLC patients. ORR was 52%, mPFS was 6 months, and 1-year OS was 88% (80). These results were comparable to the previously published ones by Gandhi et al., which showed an mPFS of 8.8 months and a 1-year OS of 69.2% (81). Additionally, a retrospective real-world POLISH study by Yang et al. showed an ORR of 28.9% and an mPFS of 5.2 months with immunochemotherapy. Notably, mPFS was not statistically significant from the chemotherapy-alone group (82).

With such data in mind, using ICI as a monotherapy in treating HER2-altered advanced NSCLC is not encouraged. However, in clinical settings, ICI in combination with chemotherapy is still considered the first-line modality for HER2 mutant NSCLC.

### HER2 monoclonal antibodies

HER2 monoclonal antibodies (mAbs) target the extracellular domain of HER2. They induce receptor downregulation and signaling inhibition, where mAbs bind to the juxtamembrane region of HER2. This prevents dimerization and activation of downstream PI3K/AKT and MAPK signaling pathways, reducing tumor cell proliferation and survival. Moreover, they could work via the antibody-dependent cellular cytotoxicity (ADCC) mechanism, engaging immune cells (e.g., natural killer cells) via their Fc region, leading to tumor cell destruction. Another mechanism of action includes the inhibition of ligand-dependent signaling, where they bind to domain II of HER2, blocking its heterodimerization with HER3 and HER1, which further inhibits the activation of growth-promoting pathways (83–87).

#### Trastuzumab

Trastuzumab is a monoclonal immunoglobulin G1 humanized murine antibody that binds to the extracellular IV domain of the HER2 receptor, blocking its dimerization, leading to receptor internalization and/or degradation, and inhibiting the PI3K/AKT signaling pathway. *In vitro* assays have also shown that Trastuzumab can activate cell-mediated cytotoxicity (88).

Trastuzumab/chemotherapy regimens are the standard in the management of advanced breast and gastric cancers with HER2 amplification or overexpression (29). Based on that, Trastuzumab/chemotherapy regimens have been tested in HER2-altered NSCLC who were previously treated. However, these studies showed variable outcomes (89).

A phase II trial assessed trastuzumab in combination with carboplatin and paclitaxel in patients with NSCLC with positive HER-2/neu Herceptest IHC results (1+ to 3+). The ORR was 24.5%, the median PFS was 3.3 months, the OS was 10.1 months, and the 1-year survival rate was 42% (90). The OS was similar to historical data using carboplatin and paclitaxel alone. However, patients with IHC3+ HER-2/neu expression did well, suggesting potential benefit for trastuzumab in this rare subset of NSCLC. However, the limited number of IHC3+ HER2 patients precludes a definitive confirmation of such results (90). Another phase II trial assessed Trastuzumab monotherapy in previously treated patients with HER2-altered NSCLC. Despite failing to report a therapeutic response (ORR = 0%), DCR was 70.0%, and the median PFS was 5.2 months (91).

#### Pertuzumab

Pertuzumab is a humanized monoclonal antibody that targets the dimerization domain (domain II) of the HER2 receptor and is considered the second most important HER2-specific monoclonal antibody after trastuzumab. By preventing HER2 from pairing with other HER family receptors, especially HER3, it inhibits downstream signaling pathways critical for tumor growth. Pertuzumab is used in combination with other HER2-targeted therapies to enhance anti-tumor efficacy (92).

In 2023, a pragmatic basket trial assessed the combination of trastuzumab and pertuzumab. It showed an ORR of 11% in heavily pre-treated patients with HER2-mutant or amplified NSCLC, particularly in those harboring HER2 exon 20 mutations (93).Additionally, in breast cancer, the combination of trastuzumab, pertuzumab, and docetaxel has shown favorable results (83, 94). Hence, the efficacy of such a combination was assessed in a single-arm phase II IFCT 1703-R2D2 trial. It included patients with HER2-mutant NSCLC previously treated with platinum-based chemotherapy (95). This combination showed an ORR of 29%, an mPFS of 6.8 months, and mDoR of 11.0 months, exceeding the duration of response observed with other HER2-targeted monoclonal antibody regimens.

### HER2 antibody-drug conjugates

ADCs are revolutionary antitumor agents that can deliver cytotoxic drugs into tumor cells expressing specific surface receptors, such as HER2. They comprise a high-activity cytotoxic payload conjugated to an anti-HER2 monoclonal antibody via a chemical linker (**Figure 1**). In addition, they can also stimulate the immune cell effector function and disrupt receptor dimerization (96). Currently, HER2-directed ACDs are considered the most effective available treatment in this context. Multiple trials have emerged in recent years with favorable outcomes (**Table 3**) (57, 97–103), with others still ongoing (**Table 4**) (104–109).

**Table 3:** Clinical outcomes with HER2 ADCs in published clinical trials for HER2-Altered NSCLC.

Drug	Trial type	Patient population	Dose	ORR (%)	mPFS (months)	mDoR (months)	OS (months)	TRAEs	Key findings
Trastuzumab-Emtansine (T-DM1)	Phase II	Relapsed HER2-altered NSCLC (15 patients)	Not specified	6.7	2	4	10.9	Elevated AST/ALT, thrombocytopenia, fatigue, nausea	Limited efficacy, early termination; only 1/7 patients with HER2-mutant achieved PR (ORR 14.3%)
	Phase II Basket Trial	HER2-mutant NSCLC (18 patients)	3.6 mg/kg IV	50	5	4	Not specified	Grade 1/2 toxicity, thrombocytopenia, fatigue, nausea	Higher ORR in HER2-mutant cohort; no grade 4 toxicities
Trastuzumab-Deruxtecan (T-DXd)	Phase I	Relapsed NSCLC with HER2 alterations (18 patients)	6.4 mg/kg IV	55.8	11.3	9.9	Not specified	ILD, fatigue, nausea, anemia, thrombocytopenia	High efficacy in HER2-altered NSCLC, especially in HER2-mutant cohort (ORR 72.7%)
	Phase II DESTINY-Lung01 Trial	HER2-mutant NSCLC (Pre-treated patients)	6.4 mg/kg IV	55	8.2	Not specified	17.8	ILD (26%), dose interruptions, discontinuations	High ORR and mPFS; ILD as a common adverse event; led to FDA approval
	Phase II DESTINY-Lung02 Trial	HER2-mutant NSCLC (Pre-treated patients)	5.4 mg/kg and 6.4 mg/kg	53.8% (5.4 mg/kg)	9.9	Not estimable	Not specified	Lower ILD and TRAE incidence with 5.4 mg/kg	5.4 mg/kg regimen outperformed 6.4 mg/kg in terms of TRAE profile and efficacy

[References: 57, 97–103].

**Table 4:** Ongoing clinical trials of HER2 ADCs in HER2-Altered NSCLC.

Drug Name	Trial Name (if available) & NCT Number	Phase	Study Type	NSCLC Population	HER2 Alteration Studied	Dosing Regimen	Primary Endpoint(s)	Key outcomes (if available)
Trastuzumab Deruxtecan (T-DXd)	DESTINY-Lung01 NCT03505710 (104)	2	Open-label, multicenter, 2-cohort study	Unresectable and/or metastatic NSCLC	HER2-overexpressing (IHC 3+ or 2+) and HER2-mutated	6.4 mg/kg or 5.4 mg/kg	Objective Response Rate (ORR)	ORR: 55% in HER2-mutant cohort; mPFS: 8.2 months
Trastuzumab Rezetecan (SHR-A1811)	DESTINY-LUNG02 NCT04644237 (105)	1/2	Open-label, dose-escalation, and expansion study	Advanced HER2-mutant NSCLC	HER2 mutations	4.8 mg/kg or 6.4 mg/kg	Safety, ORR	ORR: 41.9% in the 4.8 mg/kg cohort; mDoR: 13.7 months
Ado-Trastuzumab Emtansine (T-DM1)	NCT02675829 (106)	2	Basket trial	Metastatic lung adenocarcinoma	HER2 amplification or mutation	Not specified	ORR, Progression-Free Survival (PFS)	ORR: 44%; mPFS: 5 months
Disitamab Vedotin (RC48-ADC)	NCT04311034 (107)	1b	Open-label, multicenter study	Advanced NSCLC	HER2 overexpression or mutation	Not specified	ORR, Safety	No results reported yet
Datopotamab Deruxtecan (Dato-DXd)	TROPION-LUNG02 NCT04526691 (108)	1b	Open-label, multicenter study	Advanced or metastatic NSCLC	various genomic alterations, including HER2 alterations	Not specified	ORR, Safety	No results reported yet
Trastuzumab Deruxtecan (T-DXd)	DESTINY-Lung03 NCT04686305 (109)	1b	Multicenter, Open-label Study	Advanced NSCLC	HER2 overexpression	Not specified	ORR, Safety	No results reported yet

[References: 104–109].

#### Trastuzumab-emtansine

T-DM1 is an anti-HER2 antibody-drug conjugate (ADC) that consists of trastuzumab linked to the cytotoxic microtubule inhibitor emtansine (DM1), a derivative of maytansine. It enters HER2-positive cells via receptor-mediated endocytosis, and within the lysosomes, the antibody component undergoes proteolytic degradation, releasing DM1 (**Figure 1**) (110).

A small phase II trial evaluating T-DM1 monotherapy in 15 patients with relapsed HER2-altered NSCLC demonstrated limited efficacy, reporting an ORR of 6.7%, an mPFS of 2 months, and an OS of 10.9 months. No responses were observed in the HER2-amplified/overexpressing subgroup, while only one of seven patients in the HER2-mutant cohort achieved a partial response (PR) (ORR=14.3%). Such limited efficacy led to the early termination of the study (97). Another phase II basket trial investigated the potential role of T-DM1 in patients with HER2-mutant NSCLC. T-DM1 was administered at a dose of 3.6 mg/kg intravenously. Eight of 18 patients achieved PR, with an mDoR of 4 months and an mPFS of 5 months (98). With the updated data to include 28 pretreated patients with HER2-mutant NSCLC, ORR was reported as 50% (99). In addition, in their latter basket trial, T-DM1 was administered to 11 patients with HER2-amplified NSCLC and showed an ORR of 55% (99). T-DM1 TRAEs were reported as grades 1 and 2. Elevated AST or ALT was the most common TRAE encountered in eight patients (44%), followed by thrombocytopenia, fatigue, and nausea in 33% of patients (98). Notably, there were no grade 4 toxicities, dose reductions, or discontinuations related to treatment (98).

Additionally, another phase II study investigated the efficacy and safety of T-DM1 in patients with previously treated advanced HER2-overexpressing NSCLC. All patients received T-DM1 3.6 mg/kg IV every 3 weeks until disease progression or unacceptable toxicity, with the primary endpoint being ORR. Their results showed no treatment responses in the IHC2+ cohort (29 patients) and 4 PRs in the IHC3+ cohort (20 patients), with an ORR of 20%. However, mPFS (2.6 vs. 2.7 months) and OS (12.2 vs. 15.3 months) were comparable in the IHC2+ and IHC3+ cohorts. Notably, 3 of the 4 PRs had HER2 gene amplification, and the authors concluded that HER2 IHC as a single parameter was insufficient as a predictive biomarker (100).

#### Trastuzumab-deruxtecan

T-DXd is a next-generation HER2-targeting ADC consisting of trastuzumab, an enzymatically cleavable peptide linker, and MAAA-1181, a novel topoisomerase I inhibitor. Its mechanism of action includes binding to topoisomerase I-DNA complexes, stabilizing them, subsequently inducing DNA double-strand breaks and apoptosis. Despite having a higher drug-to-antibody ratio (8 vs. 2–4) than other ADCs, T-DXd’s stable and uniform structure ensures consistent delivery of the topoisomerase I inhibitor even in HER2-low-expressing cells. Its high membrane permeability also enables diffusion into HER2-negative cells (**Figure 1**) (111–113).

With the excellent antitumor activity of T-DXd, both *in vitro* and *in vivo*, against T-DM1-resistant and HER2-low expression models (112), a phase I trial started to evaluate T-DXd in non-breast and non-gastric/gastroesophageal tumors, enrolling 18 patients with relapsed NSCLC harboring HER2 alterations. The maximum tolerated dose was IV 6.4 mg/kg every 3 weeks. The ORR in patients with HER-altered NSCLC was 55.8%. The mDoR was 9.9 months, and the mPFS was 11.3 months. Notably, in a subgroup of 11 patients with HER2-mutant pre-treated NSCLC, the ORR was 72.7% (101, 102). Consequently, the phase II DESTINY-Lung01 trial aimed to assess T-DXd efficacy and safety at 6.4 mg/kg doses in patients with recurrent or refractory NSCLC and a HER2-mutant or overexpressed status. In the HER2-mutant subgroup receiving T-DXd monotherapy, ORR was 55%, mPFS 8.2 months, and OS 17.8 months (103). The incidence of TRAEs with T-DXd was 97%, with 46% being grade ≥ 3, leading to dose interruption in 53.1% of patients, reduction in 34.7%, or treatment discontinuation in 22.4%. Interstitial lung disease (ILD), being a main adverse effect, affected 26% of patients, leading to two deaths (103). In the following phase II DESTINY-Lung02 trial, the 5.4 mg/kg T-DXd regimen every 3 weeks outperformed the 6.4 mg/kg regimen every 3 weeks in patients with HER2-mutant and pre-treated NSCLC. The reduced dosage regimen showed higher ORR when compared to the higher dosage regimen (53.8% vs. 42.9%). The mDoR was 16.8 months (95% CI, 6.4 to not estimable [NE]) and NE (95% CI, 8.3 to NE) with the 5.4 and 6.4 mg/kg regimens, respectively. The mPFS was 9.9 months (95% CI, 7.4 to NE) and 15.4 months (95% CI, 8.3 to NE), in the 5.4 mg/kg and 6.4 mg/kg arms, respectively. Additionally, TRAEs incidence was lower in the reduced dosage regimen (31.7% vs. 58%), as was ILD (5.9% vs. 14%) (57).

Based on these results from the DESTINY-Lung02 trial, in August 2022, the FDA granted accelerated approval to T-DXd ADC in second line, HER2-mutant lung cancer, making it the first approved targeted therapy for HER2-mutant NSCLC (103). Additionally, more recently, in 2024, based on the DESTINY-PanTumor02-trial results, T-DXd received accelerated FDA approval for adult patients with unresectable or metastatic HER2-positive (IHC3+) solid tumors who have received prior systemic treatment and have no satisfactory alternative treatment options, making it the first agnostic HER2-targeted ADC, regardless of the tumor type (114). T-DXd is also under investigation as a first-line treatment in the open-label, randomized, multicenter, phase III DESTINY-Lung04 trial (NCT05048797). It aims to assess the efficacy and safety of T-DXd as a single agent compared to chemotherapy plus pembrolizumab as a first-line treatment in patients with unresectable, locally advanced, or metastatic NSCLC harboring HER2 exon 19 or 20 mutations (115, 116).

## Clinical guidelines for the treatment of advanced NSCLC with ERBB2 (HER2) mutations

NCCN Guidelines (Version 3.2025) for Non-Small Cell Lung Cancer (48) recommends starting systemic therapy based on the histologic type of NSCLC, whether adenocarcinoma or squamous cell carcinoma. This initial decision helps ensure that the treatment approach aligns with the biological characteristics of the tumor. Treating physicians should then evaluate the tumor response to treatment. This evaluation typically involves imaging studies and clinical assessments to determine if the tumor has shrunk, remained stable, or progressed. In case of a favorable tumor response or a stable disease, systemic therapy should be continued for 4 to 6 cycles before reevaluating. This period allows enough time to assess whether the treatment is effectively controlling the disease. Later on, maintenance therapy should be started in case the patient continues to have a tumor response or a stable disease. Maintenance therapy aims to prolong disease control and delay progression after initial successful treatment.

At any point in the management protocol of HER2-mutant NSCLC, if the patient experiences disease progression, subsequent therapy with ADCs should be started. Fam-Trastuzumab Deruxtecan-nxki (T-DXd) is preferred, and Ado-Trastuzumab Emtansine (T-DM1) can be an acceptable alternative.

## Management of HER2-altered NSCLC with brain metastases

In patients with HER2-altered NSCLC and brain metastases, local therapies such as stereotactic radiosurgery (SRS), whole-brain radiotherapy (WBRT), and neurosurgical resection play crucial roles in conjunction with systemic treatments. SRS is preferred for patients with a limited number of brain metastases (typically 1–4 lesions) and a good performance status, offering effective local control while preserving cognitive function more effectively than WBRT. For larger lesions (>3–4 cm) or those causing significant mass effect, surgical resection followed by postoperative SRS to the surgical cavity is recommended to enhance local control and reduce the risk of recurrence. WBRT is generally reserved for patients with extensive brain involvement or when other modalities are unsuitable, though its use is limited due to potential neurocognitive side effects. Multidisciplinary evaluation is essential for tailoring local therapy decisions based on factors such as lesion size, number, location, and patient condition. Integrating these local treatments with CNS-penetrant systemic therapies, such as T-DXd, may offer a comprehensive approach to managing CNS involvement in HER2-mutant NSCLC (117–122).

The occurrence of brain metastases in patients with HER2-altered NSCLC is a significant clinical challenge, with reported incidence rates ranging from 6% to 29% (26, 123, 124). Among the various HER2 mutations, the exon 20 YVMA insertion has been particularly associated with a markedly higher baseline and lifetime risk of developing brain metastases (125). This underscores the aggressive nature of this mutation and the need for effective treatment strategies. The central nervous system (CNS) remains a common site for metastases due to the restrictive nature of the blood-brain barrier (BBB), which limits the penetration of many systemic therapies, including conventional chemotherapeutic agents and targeted therapies. As a result, patients with HER2-altered NSCLC and CNS involvement often experience disease progression despite systemic treatment, leading to poorer overall prognosis and survival outcomes (126).

Although Pan-HER TKIs such as afatinib, dacomitinib, and pyrotinib have demonstrated systemic efficacy in HER2-mutant NSCLC, their impact on the CNS remains unclear due to limited available data. In contrast, poziotinib has shown a 28.6% ORR and an mPFS of 7.4 months in patients with brain metastases. However, the reliability of these findings is limited by the small sample size, the absence of a baseline brain MRI, and the frequent use of prior brain radiation (59).

In the phase III DESTINY-Breast03 trial, T-DXd has demonstrated notable intracranial efficacy in HER2-positive breast cancer. Patients with stable brain metastases at baseline experienced an impressive intracranial response rate of 63.8% with T-DXd, compared to only 33.3% with T-DM1 (127). Additionally, a subgroup analysis from the DESTINY-Breast01 study showed T-DXd’s effectiveness in treating stable brain metastases in individuals previously treated with T-DM1 (128). T-DXd has also shown promising results in patients with active brain metastases from HER2-positive metastatic breast cancer. The phase II DEBBRAH trial reported an intracranial response rate of 44.4% in patients with either HER2-positive or HER2-low breast cancer who experienced brain metastases progression after local therapy (129). Furthermore, in the phase II TUXEDO-1 trial, T-DXd achieved an intracranial response rate of 100% in patients with newly diagnosed brain metastases and 66.7% in those with progressive brain metastases (130).

Similar to T-DXd’s demonstrated efficacy in HER2-positive breast cancer, it has also shown promising intracranial activity in patients with HER2-mutant NSCLC. A pooled analysis of data from the DESTINY-Lung01 and DESTINY-Lung02 trials revealed significant reductions in brain lesion size among patients with measurable brain metastases. Specifically, 86% of those receiving a 5.4 mg/kg dose and 78% of those receiving a 6.4 mg/kg dose of T-DXd experienced a decrease in the size of their brain metastases (13, 57, 103). These findings suggest that T-DXd may play a crucial role in addressing CNS involvement in HER2-mutant NSCLC, a population that often faces limited treatment options due to the challenges posed by the blood-brain barrier. Further research is underway to better define the T-DXd efficacy in this setting. The ongoing phase III DESTINY-Lung04 trial (NCT05048797) aims to evaluate CNS PFS as a secondary endpoint, providing valuable insights into the durability of T-DXd’s CNS activity (115, 116). The results of this trial will be instrumental in determining the long-term impact of T-DXd on brain metastases in patients with HER2-mutant NSCLC and may further support its role as a key therapeutic option in this patient population.

At the 2024 World Conference on Lung Cancer (WCLC), Opdam et al. presented a subanalysis of phase 1b of the Beamion LUNG-1 trial focusing on zongertinib’s efficacy in patients with HER2-positive solid tumors and baseline brain metastases. This analysis included 132 patients with advanced or metastatic HER2-mutant non-small cell lung cancer (NSCLC), 41% of whom had asymptomatic brain metastases at baseline. Patients with brain metastases exhibited an ORR of 70%, comparable to the 73% ORR observed in patients without brain metastases. The DCR was 94% in patients with brain metastases and 96% in those without, indicating consistent disease stabilization across both groups. Among patients with brain metastases, the intracranial ORR was 37%, with a DCR of 83%. Notably, 17% achieved complete intracranial responses, demonstrating zongertinib’s significant effectiveness in controlling central nervous system lesions (131).

## Future directions of HER2-altered NSCLC

The treatment landscape for HER2-driven NSCLC is rapidly evolving, shifting from monotherapy to combination targeted therapies that leverage synergistic effects. This transition is expected to enhance treatment efficacy and provide more durable responses for patients with HER2-altered NSCLC.

### Novel HER2-directed TKIs

**NVL-330** is a novel, brain-penetrant, HER2-selective TKI targeting HER2-altered tumors, including those with HER2 exon 20 insertion mutations. Its design aims to avoid off-target inhibition of wild-type EGFR and effectively address brain metastases (132). In preclinical studies, NVL-330 exhibited broad activity against various HER2 oncogenic alterations, such as HER2 exon 20 insertions, activating point mutations, and amplified wild-type HER2 (132). In July 2024, the HEROEX-1 phase 1a/1b (NCT06521554) clinical trial was initiated to evaluate NVL-330 in pre-treated patients with advanced HER2-altered non-small cell lung cancer (NSCLC). The trial aims to assess the safety, tolerability, pharmacokinetics, and preliminary anti-tumor activity of NVL-330, as well as determine the recommended phase 2 dose (71).

BAY 2927088 is an investigational oral, reversible TKI, designed to selectively target mutant forms of HER2 and EGFR, particularly in NSCLC harboring HER2-activating mutations (68). In the phase I/II SOHO-01 trial, patients with advanced NSCLC harboring a HER2-activating mutation who experienced disease progression after at least one systemic therapy but were naïve to HER2-targeted therapy were enrolled and received BAY 2927088 at 20 mg twice daily. Overall, 34 pts were treated, with a median follow-up of 8 months. Study results showed mPFS of 8.1 months (95% CI: 4.4–not evaluable), ORR of 70% (95% CI: 51.3–84.4), and DCR of 82% (95% CI: 64.5–93.0). In these patients, 10 patients had a dose reduction, 8 had dose interruptions, and 3 discontinued study treatment due to TRAEs. The most common adverse events were diarrhea (85%; mainly grade 1-2) and rash (47%; grade 1-2) (68). The phase III SOHO-02 trial was initiated in early 2024. This open-label, randomized, multicenter trial aims to assess the efficacy and safety of BAY 2927088 as a first-line therapy in patients with locally advanced or metastatic NSCLC with HER2-activating mutations (69). Additionally, in February 2024, the FDA granted breakthrough therapy designation to BAY 2927088 for the treatment of adult patients with unresectable or metastatic NSCLC whose tumors have activating HER2 mutations and who have received prior systemic therapy (70). This underscores the potential of BAY 2927088 as a targeted therapy for patients with HER2-mutant NSCLC, addressing a significant unmet medical need in this population.

### Combinations of ADCs with ICIs

One promising approach is the combination of ADCs with ICIs, which has shown potential in preclinical and clinical studies (133). The combination of ADCs with ICIs is biologically compelling due to ADCs’ ability to induce immunogenic cell death (ICD), leading to increased tumor antigen presentation and immune cell recruitment. Preclinical studies have shown that trastuzumab deruxtecan (T-DXd) enhances PD-L1 and MHC-I expression and promotes CD8+ T-cell infiltration in the tumor microenvironment, suggesting potential synergy with ICIs (134–136). Moreover, the bystander effect of T-DXd can further facilitate immune activation by releasing cytotoxic payloads into surrounding tumor tissue (112). These mechanisms support ongoing clinical investigations evaluating ADC–ICI combinations in HER2-altered NSCLC (135). Preclinical research has demonstrated that T-DXd can increase the expression of PD-L1 via major histocompatibility complex class I (MHC-I) and promote the infiltration of CD8+ T cells into tumor cells. These findings suggest that combining T-DXd with ICIs could enhance antitumor immune responses (133).

In light of this, several clinical trials are exploring this combination. The phase II HUDSON basket trial investigated the combination of T-DXd and Durvalumab in patients previously treated with anti-PD1/PD-L1 therapy, including those with HER2-mutant (HER2m) and HER2-overexpressed (HER2e) NSCLC. The results indicated that patients with HER2-mutant NSCLC experienced more significant benefits from this combination (135). The ORR was 35% (80% CI, 20.7–51.8) in the HER2m group, compared to 26.1% (80% CI, 14.3–41.3) in the HER2e group. The mPFS was 5.7 months (80% CI, 5.5–6.5) in the HER2m group, whereas in the HER2e group was 2.8 months (80% CI, 2.2–5.5). The mOS was 10.6 months (80% CI, 8.9–not calculable) for the HER2m group and 9.5 months (80% CI, 6.6–12.4) for the HER2e group. Additionally, Grade ≥3 TEAEs occurred in 50% of patients in the HER2m group and 61% in the HER2e group. Treatment-related pneumonitis was observed in 10% of HER2m patients and 8.7% of HER2e patients; however, no fatal events were reported.

Additionally, the ongoing phase Ib DESTINY-Lung03 trial (NCT04686305) is evaluating the safety, tolerability, and efficacy of T-DXd in combination with Durvalumab and chemotherapy as a first-line treatment in advanced HER2-overexpressed NSCLC (109). Another active phase Ib study (NCT04042701) is assessing the combination of T-DXd and ICI, pembrolizumab, in HER2-mutant and HER2-positive NSCLC patients who have not previously received HER2-targeted or anti-PD-1/PD-L1 therapies (137).

Despite these combination therapies’ potential benefits, they may pose challenges. Both T-DXd and Durvalumab have been associated with pulmonary complications. In the HUDSON trial, 55% of patients experienced grade ≥3 TEAEs, including pneumonitis, pulmonary embolism, and anemia, with pneumonitis being the most frequently observed adverse event. All grades and grades≥3 treatment-related pneumonitis occurred in 9.3% and 7% of all patients, respectively (135). This underscores the need for careful patient selection and close monitoring during treatment.

### Combinations of ICIs with TKIs

Another therapeutic approach is ICIs in combination with TKIs. This combination is currently being evaluated for HER2-mutant NSCLC after first-line chemotherapy failure. A phase II study (NCT04144569) is investigating pyrotinib in combination with PD-1 inhibitors as second-line treatment, based on the hypothesis that TKIs induce immunogenic cell death and cytokine release, thereby enhancing tumor antigen presentation and immune activation (138). In parallel, ICIs help sustain the immune response by preventing T-cell exhaustion, leading to a more effective antitumor effect (24).

### Novel HER2-targeted ADCs

Another emerging field of HER2-directed pharmacology focuses on optimizing ADCs’ design to improve efficacy while minimizing toxicity. For instance, such novel HER2-targeted ADCs include A166, ARX788, SHRA1811, and MRG002. These structural advancements represent a new generation of ADCs aiming to maximize therapeutic indices and overcome resistance mechanisms seen with earlier HER2-targeted therapies.

A166 is an ADC consisting of a cytotoxic drug (duostatin-5) site-specifically conjugated to a humanized anti-HER2 antibody (trastuzumab) and bound to a stable protease-cleavable valine citrulline linker, allowing efficient intracellular drug release (139, 140). A phase I study showed that A166 demonstrated clinically meaningful efficacy in heavily pretreated patients with relapsed or refractory advanced solid cancers, with patients achieving an ORR of 36% at efficacious dose levels and up to 100% in HER2 positive patients regardless of histology (2 CRC, 1 BC and 1 NSCLC) at the highest studied dose level (141). Another Phase I study evaluated A166 in patients with advanced solid tumors, primarily metastatic breast cancer, who had previously received anti-HER2 therapies. For all assessable HER2-positive breast cancer patients enrolled in the 4.8 mg/kg cohort, the ORR was 73.9%, and the mPFS was 12.3 months (140). Such studies provide insights into the potential of A166 as a therapeutic option for patients with HER2-altered NSCLC.

SHRA1811 is another novel ADC, trastuzumab-rezetecan. It is engineered with a topoisomerase I inhibitor payload and a novel linker system that minimizes premature cleavage and off-target toxicity, contributing to its favorable safety profile and antitumor activity in HER2-mutant NSCLC (142, 143). A dose-escalation and expansion, multicenter, open-label, phase 1/2 study (NCT04818333) was conducted to assess SHR-A1811 in pretreated HER2-altered advanced NSCLC. The total number of patients enrolled was 63, all with HER2-mutant disease. In the 4.8 mg/kg cohort, ORR was 41.9%, DCR was 95.3%, mDoR was 13.7 months, and mPFS was 8.4 months. All patients experienced at least any grade of TRAEs, with grade ≥3 reported in 46% of patients. The most common grade ≥3 TRAEs were decreased neutrophil count, white blood cell count, and anemia in 30.2%, 22.2%, and 14.3%, respectively. With such results, SHR-A1811 demonstrated favorable safety and clinically meaningful efficacy in pretreated advanced HER2-mutant NSCLC (144). Furthermore, a phase Ib/II trial (NCT05482568) is currently recruiting to assess SHR-A1811 in combination with pyrotinib or SHR-1316, an anti-PD-L1 antibody, in patients with advanced HER2-altered NSCLC (143).

Other novel ADCs that may have therapeutic potential in HER2-mutant NSCLC include ARX788, which was tested in a phase I trial for the treatment of HER2-positive metastatic breast cancer (145), and MRG002, which was tested in a phase I dose escalation and expansion study in patients with HER2-positive solid tumors (146). ARX788 utilizes a non-cleavable linker and a potent tubulin-inhibitory cytotoxin (AS269), conjugated site-specifically via a proprietary amber suppression technology that enables a precise drug-to-antibody ratio for improved pharmacokinetics and reduced immunogenicity (145). Similarly, MRG002 is built with a cleavable linker and monomethyl auristatin E (MMAE) payload, designed to ensure selective HER2 targeting and cytotoxicity while mitigating systemic exposure (146).

SYD985 is another HER2 ADC, consisting of trastuzumab conjugated to a duocarmycin-based payload (T-Duo) via a cleavable valine-alanine linker, which remains stable in circulation but is selectively cleaved by tumor-associated proteases. This design allows for efficient intracellular release of the cytotoxic agent and supports a potent bystander effect, enhancing efficacy in tumors with heterogeneous or low HER2 expression (147, 148). The NCT04235101 trial explores the safety of combining SYD985 with the PARP inhibitor niraparib in patients with solid tumors. To the best of our knowledge, and as of January 2024, the trial has been completed, with 32 participants enrolled. However, study results regarding this combination safety profile, efficacy outcomes, and recommended dosing schedules have not been publicly disclosed (147). It is also worth mentioning that T-Duo was compared with the physician’s choice (PC) standard treatment in a randomized, international, multicenter, phase 3 study in 231 patients with pre-treated HER2-positive metastatic breast cancer (148). Median OS was 21.0 months in the T-Duo group and 19.5 months in the PC group. The 1-year survival estimate was 70% in the T-Duo group and 68% in the PC group. The primary endpoint mPFS was 7.0 vs 4.9 months, respectively (p=0.002), and other secondary efficacy outcomes did not change in this analysis compared to the initial analysis (148).

### Liquid biopsy in monitoring treatment efficacy

Emerging evidence also highlights the role of liquid biopsy in monitoring treatment efficacy. Recent studies have shown that fluctuations in ctDNA levels correlate with treatment outcomes and survival rates in solid tumors undergoing targeted therapy (149). A recent analysis of HER2-mutant NSCLC patients receiving pyrotinib from two phase II clinical trials reported better treatment responses in those who achieved ctDNA clearance after 40 days of therapy. However, the small sample size necessitates further validation (150). Additionally, a case report demonstrated the utility of ctDNA analysis by showing that changes in HER2 mutation allele frequency in plasma closely mirrored the clinical course of a patient undergoing T-DXd treatment (151). Furthermore, recent developments in ctDNA-based testing have shown potential for detecting HER2-amplified tumors, which could improve the clinical application of HER2-targeted therapies. A multi-center Phase-II Basket Trial highlighted that ctDNA detection of HER2 amplification could help identify patients likely to benefit from T-DXd, particularly in cases where tissue biopsies are impractical (152).

## Conclusion

HER2-altered NSCLC represents a critical area of exploration for novel therapeutic strategies, offering significant challenges due to the limited treatment options available for patients with these mutations. Over the years, understanding the molecular landscape of HER2 in NSCLC has evolved, leading to the development of targeted therapies such as ADCs like T-DXd and combinations with ICIs and TKIs, and the emergence of novel HER2-specific, better-tolerated, and more efficacious TKIs such as zongertinib. These treatments have shown promising efficacy, including patients with brain metastases, which cause significant morbidity and mortality in patients with HER2-driven NSCLC.

Clinical trials exploring these novel combinations are ongoing in HER2-mutant NSCLC, with encouraging results from studies like the DESTINY-Lung trials, zongertinib Beamion LUNG-1 studies, and other phase I/II studies testing the synergy between HER2-targeted therapies and ICIs. Liquid biopsy, including ctDNA analysis, is emerging as an essential tool to monitor treatment efficacy and progression, offering a non-invasive alternative to traditional biopsy methods.

While challenges such as pulmonary toxicity from combination therapies and the need for optimized treatment regimens remain, the future of HER2-targeted therapies in NSCLC is promising. As these therapies continue to show efficacy in preclinical and early clinical trials, it is crucial to continue advancing research to improve patient outcomes. Personalized medicine, based on molecular profiling and biomarkers like HER2, is likely to shape the next generation of treatments for this patient population, offering new hope for those battling this aggressive form of lung cancer.
